# Developing Targeted Therapies That Exploit Aberrant Histone Ubiquitination in Cancer

**DOI:** 10.3390/cells8020165

**Published:** 2019-02-16

**Authors:** Lucile M-P Jeusset, Kirk J McManus

**Affiliations:** 1 Department of Biochemistry & Medical Genetics, University of Manitoba, Winnipeg, MB R3E 0J9, Canada; jeussetl@myumanitoba.ca; 2 Research Institute in Oncology and Hematology, CancerCare Manitoba, Winnipeg, MB R3E 0V9, Canada

**Keywords:** histone, H2A, H2B, ubiquitination, deubiquitination, E3 ubiquitin ligase, cancer, epigenetic therapy, synthetic lethality, epigenome editing

## Abstract

Histone ubiquitination is a critical epigenetic mechanism regulating DNA-driven processes such as gene transcription and DNA damage repair. Importantly, the cellular machinery regulating histone ubiquitination is frequently altered in cancers. Moreover, aberrant histone ubiquitination can drive oncogenesis by altering the expression of tumor suppressors and oncogenes, misregulating cellular differentiation and promoting cancer cell proliferation. Thus, targeting aberrant histone ubiquitination may be a viable strategy to reprogram transcription in cancer cells, in order to halt cellular proliferation and induce cell death, which is the basis for the ongoing development of therapies targeting histone ubiquitination. In this review, we present the normal functions of histone H2A and H2B ubiquitination and describe the role aberrant histone ubiquitination has in oncogenesis. We also describe the key benefits and challenges associated with current histone ubiquitination targeting strategies. As these strategies are predicted to have off-target effects, we discuss additional efforts aimed at developing synthetic lethal strategies and epigenome editing tools, which may prove pivotal in achieving effective and selective therapies targeting histone ubiquitination, and ultimately improving the lives and outcomes of those living with cancer.

## 1. Introduction

Epigenetics is the regulation of DNA-driven processes, such as gene transcription, DNA replication, and DNA damage repair, by chromatin modifications other than changes to the DNA sequence [1]. Epigenetic mechanisms include chromatin remodeling, DNA methylation, and histone post-translational modifications, as well as the regulation of these chromatin alterations by non-coding RNAs, including microRNAs and long non-coding RNAs. Epigenetic regulation of gene expression normally governs development, cellular differentiation, and maintenance of stem-cell populations in adult tissues [2,3,4]; however, epigenetic aberrations occur in virtually all cancers and are proposed to drive oncogenesis, at least in part by repressing tumor suppressor genes and/or activating oncogenes [1,2]. Conceptually, as epigenetic alterations are inherently reversible, the therapeutic targeting and reversal of the aberrant epigenetic mechanisms in cancer cells is predicted to restore normal epigenetic regulation of gene expression, promote cell differentiation, halt cellular proliferation, and induce cell death.

For over two decades, cancer epigenetics research and therapeutic strategies focused predominantly on targeting aberrant DNA methylation and histone methylation/acetylation. To date, two DNA hypomethylating agents (azacitidine, decitabine) and four histone deacetylase inhibitors (vorinostat, romidepsin, belinostat, panobinostat) received Food and Drug Administration (FDA) approval as second- or third-line chemotherapies for certain hematological malignancies [5,6,7,8,9]. Clinical development is now focused on evaluating the relevance of these epigenetic agents for the treatment of solid tumors, as well as developing novel therapeutic strategies that target the proteins recruited to the epigenetic/chromatin modifications (known as “readers”) [9,10]. In parallel, there is an emerging interest in therapeutically targeting the machinery responsible for the addition (“writers”) or removal (“erasers”) of additional histone modifications, such as histone ubiquitination. Histone ubiquitination is of particular interest, as aberrant ubiquitination drives oncogenesis by altering the expression of key tumor suppressors and oncogenes and promoting cancer cell proliferation [11,12,13,14,15,16,17]. Therefore, there is growing impetus to develop therapies that target aberrant histone ubiquitination to induce death in cancer cells. We begin this review by first defining the normal functions ascribed to histone ubiquitination, before discussing ongoing strategies aimed at reversing aberrant histone ubiquitination, including both their benefits and limitations. Finally, we end with a discussion of three key research areas that are predicted to enable the development of histone ubiquitination-based therapies with enhanced selectivity and efficacy in cancer.

## 2. Histone Ubiquitination Regulates Gene Transcription and DNA Damage Repair

**Figure 1:**
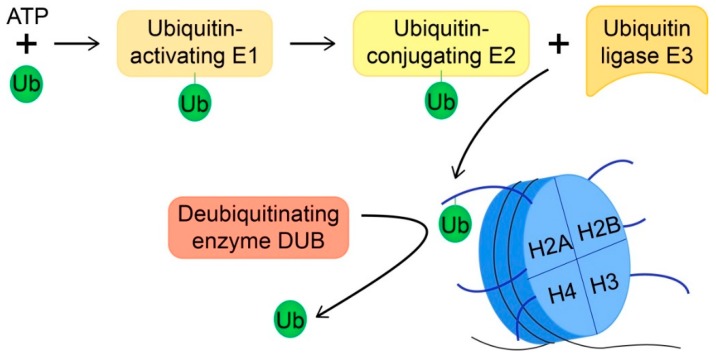
Schematic presenting the enzymatic machinery catalyzing the addition and removal of histone ubiquitination. Ubiquitination requires a ubiquitin-activating enzyme (E1), a ubiquitin-conjugating enzyme (E2), and a ubiquitin ligase (E3). As depicted, histones are typically ubiquitinated on lysine residues contained within histone tails that extend away from the nucleosome. Ubiquitin (Ub) is removed from histones (i.e., target proteins) by a deubiquitinating enzyme (DUB).

Ubiquitin is an 8.5-kDa polypeptide consisting of a globular domain and a flexible six-amino-acid carboxy-terminal tail terminating with a glycine residue. Ubiquitination is the formation of an isopeptide bond between this terminal glycine and the ε-amino group of a lysine residue from the target protein [18,19]. The addition of a ubiquitin moiety to a target protein is catalyzed by the successive actions of three enzymes (Figure 1). Ubiquitin is first loaded onto a ubiquitin-activating enzyme (E1), in an ATP-dependent reaction, before being transferred to a ubiquitin-conjugating enzyme (E2), and finally ligated to the target lysine residue by a ubiquitin ligase (E3) [18,20]. E3 ubiquitin ligases are primarily responsible for substrate specificity [18]. Target proteins may be monoubiquitinated or polyubiquitinated, with chains of two to ten ubiquitin molecules covalently linked via one of the seven lysine residues contained in the ubiquitin molecule or its N-terminal methionine [19]. K48-linked polyubiquitin chains generally target proteins for proteasomal degradation via the 26S proteasome, while monoubiquitin and K63-linked polyubiquitin chains typically modulate protein function, localization, or interaction with DNA and/or other proteins [19]. Protein ubiquitination levels are dynamically regulated by the opposing activity of deubiquitinating enzymes (DUBs), also known as ubiquitin-specific peptidases (USPs).

In vivo, all four core histones H2A, H2B, H3, and H4, and the linker H1 may be mono- or polyubiquitinated on multiple lysine residues [21]. These dynamic post-translational modifications control gene transcription and DNA damage repair through multiple mechanisms, including regulating histone–DNA interaction, nucleosome stability, histone eviction, chromatin compaction, histone cross-talk, or recruitment of reader proteins [22,23,24,25,26]. Unfortunately, the majority of histone ubiquitination events (i.e., specific lysine residue coupled with specific ubiquitination topology) are yet to be ascribed a biological function, particularly those found on histones H1, H3, and H4. For instance, while ring finger protein 8 (RNF8)-mediated H1 polyubiquitination plays an important role in the repair of DNA double-strand breaks (DSBs), it remains unclear which H1 lysine residues are involved in this process [27]. Similarly, while H3 and H4 ubiquitination may participate in several DNA damage repair pathways, the relevant ubiquitination sites remain to be identified [22,28]. Accordingly, this review focuses on the ubiquitination of histones H2A and H2B, whose functions and regulation are better understood (Table 1 and Table 2).

Histone H2A can be ubiquitinated on lysine residues K13, K15, K119, K125, K127, and K129 in vivo [21]. K119 is the most frequently observed ubiquitination site, and monoubiquitinated K119 (H2AK119ub1) occurs on approximately 10% of all nucleosomal H2As [29,30,31,32]. The predominant E3 ubiquitin ligase for H2AK119ub1 is the catalytic subunit of the polycomb repressive complex (PRC1), composed of really interesting new gene 1A (RING1A) and RING1B, and activated by B-lymphoma Moloney murine leukemia virus insertion region 1 homolog (BMI1) [33,34,35]. The major H2AK119ub1 DUBs are USP16 and breast cancer type 1 susceptibility protein (*BRCA1)*-associated protein 1 (BAP1) [36,37]. H2AK119ub1 is enriched within promoter regions of polycomb target genes and functions as a transcriptional repressor through a variety of mechanisms (Table 1) [38]. H2AK119ub1-mediated repression of polycomb target genes is necessary for the maintenance of stem-cell populations within adult tissues, including intestinal and hematopoietic stem cells, and dynamic regulation of H2AK119ub1 by USP16 and BMI1 controls normal hematopoiesis [37,39,40,41,42]. In addition, H2AK119ub1 is involved in DNA damage repair, as DSBs induce localized BMI1-mediated monoubiquitination of K119, which represses transcription of the break-flanking regions and promotes repair by homologous recombination [43,44,45,46]. In addition to H2AK119ub1, H2A ubiquitination also occurs on additional lysine residues in response to DNA damage. For example, RNF168-mediated monoubiquitination of H2AK15 is required for the recruitment of tumor protein 53 (TP53)-binding protein 1 (53BP1) to DSBs, while H2AK15 polyubiquitination (K63 linkage) by RNF168 recruits the BRCA1-A complex. Both 53BP1 and the BRCA1-A complex modulate DNA end resection [27,47,48,49,50]. In addition, BRCA1 is an important E3 ubiquitin ligase that monoubiquitinates H2AK127 and/or H2AK129 to recruit the SWI/SNF-related, matrix-associated actin-dependent regulator of chromatin, subfamily A, containing DEAD/H box 1 protein (SMARCAD1), a chromatin remodeling protein that promotes DNA end resection and homologous recombination [29,51].

As with H2A, histone H2B is ubiquitinated on multiple lysine residues in vivo, including K34 and K120. H2BK120 monoubiquitination (H2BK120ub1) is the most abundant form of H2B ubiquitination, present on approximately 1% of all nucleosomal H2Bs [32,66], and its addition is catalyzed predominantly by the E3 ubiquitin ligase complex RNF20/40 [67,68]. USP22, which is ubiquitously expressed in human tissues, is arguably the best characterized H2BK120ub1 DUB [69,70,71]. However, at least eight other DUBs can deubiquitinate H2BK120ub1 in human cells (Table 2). Although certain DUBs may be partially redundant [72], ongoing characterization of these enzymes indicate that they associate with distinct complexes, target different regions of the genome, and function in distinct pathways and cellular processes (Table 2). In addition, differences in tissue expression levels suggest that H2BK120ub1 DUBs may have tissue-specific functions [71]. To date, the complex composed of male-specific lethal 1 homolog (MSL1) and MSL2 is the only E3 ubiquitin ligase known to catalyze H2BK34 monoubiquitination (H2BK34ub1) [66,73] and a corresponding DUB is yet to be identified. H2BK120ub1 and H2BK34ub1 are co-dependent, as H2BK34ub1 promotes the recruitment of RNF20/40 and subsequent H2BK120 ubiquitination, while H2BK120ub1 promotes the recruitment of MSL1/MSL2 and subsequent H2BK34 ubiquitination [66,73]. In general, both H2BK120ub1 and H2BK34ub1 promote gene transcription (Table 2), while H2BK120ub1 is also necessary for efficient DSB repair and recruiting effectors for the two major DSB repair pathways, non-homologous end-joining and homologous recombination repair [25,74]. Remarkably, USP22-mediated H2BK120ub1 deubiquitination is also critical for efficient DSB repair through both of the major repair pathways [75,76], indicating that H2BK120ub1 levels must be dynamically regulated for efficient DSB repair.

## 3. Targeting Aberrant Histone Ubiquitination in Cancer

A large number of immunohistochemical analyses revealed that aberrant histone ubiquitination patterns exist in many cancer types. For example, global decreases in H2AK119ub1 levels occur in prostate cancer, while decreased H2BK120ub1 levels are frequently observed in breast, lung, and colorectal cancers relative to normal tissues [57,88,89,90]. Moreover, DNA- and RNA-sequencing data show that the genes encoding histone E3 ubiquitin ligases and DUBs are also frequently altered in cancers [91], and many of the enzymes possess tumor suppressor (e.g., BAP1 and RNF20) or oncogenic potential (e.g., BMI1 and USP22) [44,70,92,93,94], identifying possible mechanism(s) accounting for the aberrant histone ubiquitination levels observed within those cancers. While the normal and cancer-associated functions of H2AK15 and H2AK127/129 ubiquitination are only beginning to emerge (Table 3), the impact that aberrant H2AK119ub1 and H2BK120ub1 have in cancer is better defined. Indeed, clinical and molecular insight into H2AK119ub1 and H2BK120ub1 in cancer led to the development of new therapeutic strategies (discussed below) that seek to reset these modifications to effectively reprogram cellular transcription, halt cellular proliferation, and preferentially induce death in cancer cells.

### 3.1. Targeting Increased H2AK119ub1 Levels and BMI1 Overexpression in Hematological and Solid Malignancies

**Figure 2:**
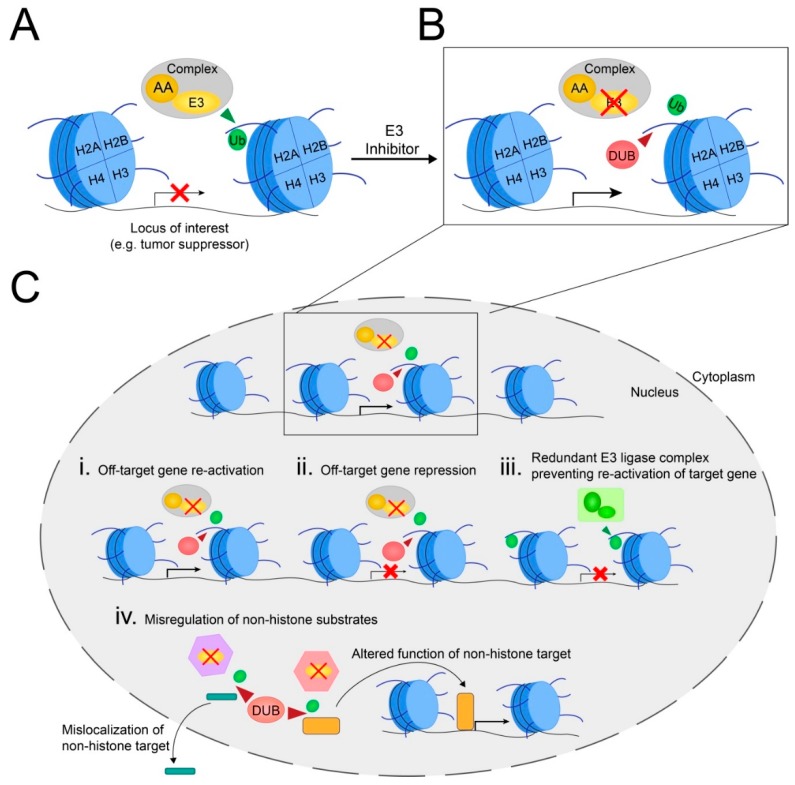
Schematic presenting putative impacts associated with targeting the histone ubiquitination machinery. (**A**) In cancer, overexpression of a histone E3 ubiquitin ligase (e.g., really interesting new gene 1A/1B (RING1A/RING1B)) or its allosteric activator (AA; e.g., B-lymphoma Mo-MLV insertion region 1 homolog (BMI1)) can repress expression of tumor suppressor genes. (**B** )Following therapeutic inhibition of an E3 ubiquitin ligase or its allosteric activator, ongoing DUB activity will remove the ubiquitin mark at the locus of interest resulting in gene derepression (i.e., gene re-activation). (**C**) Inhibition of the E3 ubiquitin ligase impacts additional processes; it may re-activate **(i)**, or repress expression of additional off-target genes **(ii)**, while other genes of interest may not be re-activated if a functionally redundant E3 ubiquitin ligase compensates for the loss of the inhibited E3 ubiquitin ligase **(iii)**. In addition, inhibition of the E3 ubiquitin ligase may deactivate additional complexes it associates with (hexagons), resulting in misregulation of ubiquitination on non-histone targets **(iv)**. This may impact their localization and function (such as activation of transcription factors) and induce further off-target effects.

The role of the polycomb E3 ubiquitin ligase subunit RING1A/RING1B/BMI1 and H2AK119ub1 in the maintenance of stem-cell populations in adult tissues suggests it may harbor a role in the maintenance of cancer stem cells. In this regard, *BMI1* is overexpressed and promotes cancer cell self-renewal in acute myeloid leukemia and several solid tumor types, such as pancreatic cancer, glioblastoma multiforme, diffuse intrinsic pontine glioma, colorectal cancer, and epithelial ovarian cancer [12,13,14,96,97,98,99,100,122]. In leukemic cells, BMI1 promotes cancer cell self-renewal via H2AK119ub1-mediated repression of key tumor suppressor genes, including the *INK4A/ARF* locus (Figure 2A) [12,13,14]. Interestingly, high expression of *BMI1, RING1A*, and/or *RING1B* correlates with worse overall survival in acute myeloid leukemia [91,122], suggesting that high H2AK119ub1 levels may be pathogenic. Collectively, these findings suggest that re-activation of key tumor suppressor genes following RING1A/RING1B/BMI1 inhibition may be a therapeutic strategy to inhibit cancer stem-cell proliferation and/or induce cell death (Figure 2B). In agreement with this possibility, several small-molecule inhibitors were developed, including the orally bioavailable compound PTC-596 that induces hyperphosphorylation and subsequent depletion of BMI1 [123]. In acute myeloid leukemia cell lines, PTC-596 decreases BMI1 and H2AK119ub1 levels and induces apoptosis, while it also prolongs survival in xenograft mouse models of acute myeloid leukemia [101]. In ovarian cancer models, PTC-596 administration induced apoptosis in ovarian cancer cell lines, and decreased tumor weight in orthotopic mouse models with an efficacy similar to that of cisplatin/paclitaxel, the current standard of care [123]. In 2015, a phase I clinical trial was carried out for adult patients with advanced solid tumors that reported manageable side effects [124]. Currently, two phase Ib trials are ongoing with PTC-596, either in combination with carboplatin/paclitaxel for patients with stage III–IV epithelial ovarian cancer receiving neoadjuvant chemotherapy, or in combination with radiation therapy for pediatric patients with high-grade glioma or diffuse intrinsic pontine glioma (Table 3). Thus, these pre-clinical findings combined with encouraging clinical study results highlight the potential utility of BMI1 inhibitors as clinically relevant therapeutic agents.

### 3.2. Exploiting Reduced H2BK120ub1 Abundance in Cancer Therapeutics

**Figure 3:**
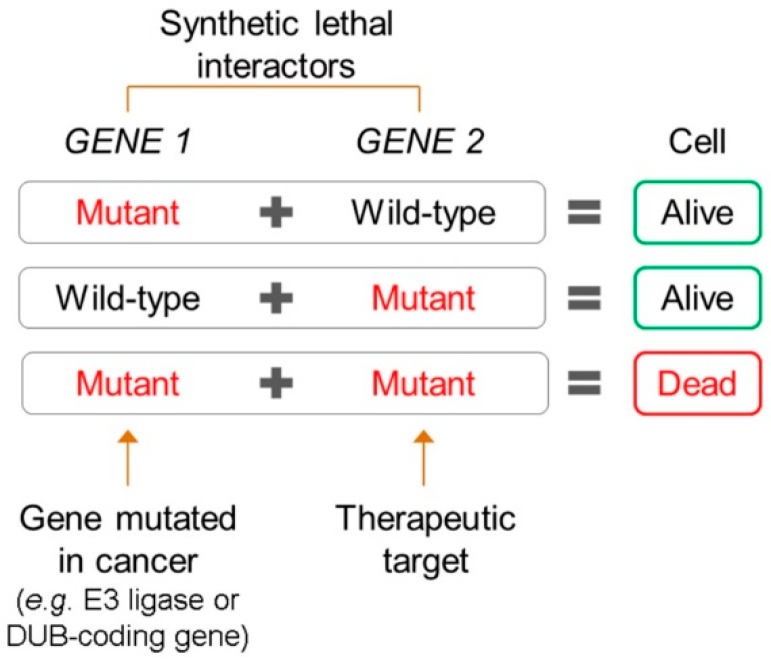
The synthetic lethal paradigm in cancer targeting. Synthetic lethality describes a rare and lethal genetic interaction occurring between two unlinked genes. Mutually exclusive mutations or alterations occurring in either *GENE 1* or *GENE 2* are viable, while simultaneous co-occurrence is lethal. In a therapeutic context, cancer cells with genetic defects in a histone E3 ubiquitin ligase or DUB gene (e.g., *GENE1*) can be selectively targeted and killed by downregulating or inhibiting the expression and/or function of a synthetic lethal interactor (*GENE 2*; drug target).

The global loss of H2BK120ub1 occurs in approximately 70% of primary breast [88] and colon cancer samples [89], and is associated with poorer patient outcomes in colon cancer [89]. Conceptually, the global depletion of H2BK120ub1 may occur as a result of reduced E3 ubiquitin ligase (e.g., RNF20/RNF40) activity and/or increased activity of the relevant DUBs (e.g., USP22). In this regard, the *RNF20* promoter is hypermethylated in breast cancer [17] and reduced expression of *RNF20* and/or *RNF40* is observed in seminoma, basal-like breast cancer, and colorectal cancer [15,111,112]. Conversely, *USP22* overexpression is associated with poor patient outcomes in multiple cancer types, including colorectal, pancreatic, breast, and epithelial ovarian cancers [89,114,115,116,117,118,119]. Reduced *RNF20* expression and the concomitant reduction in H2BK120ub1 levels are known to activate the expression of key proto-oncogenes (e.g., *MYC* and *FOS*) and, therefore, may be significant drivers of oncogenesis [17]. In addition, depletion of RNF20 and H2BK120ub1 induces replication stress, adversely impacts DNA damage repair, and underlies chromosome instability, aberrant phenotypes that all contribute to cancer development and progression [111,125,126,127]. Furthermore, USP22 is a transcriptional activator which cooperates with several oncoproteins (e.g., c-MYC and the androgen receptor) to activate expression of their target genes following H2BK120ub1 deubiquitination [80,128]. Consequently, USP22 is frequently hailed as an attractive target for cancer therapeutics [89,114,115,116,117,118,119]. Unfortunately, however, a USP22-specific inhibitor remains to be identified and, thus, an attractive alternative may be to employ synthetic lethality, a strategy that selectively targets cancers with genetic defects in cancer-promoting genes. In genetic terms, synthetic lethality defines a rare and lethal combination of two independently viable mutations (Figure 3) and can be levied against cancer cells harboring specific alterations in key genes (e.g., *RNF20* deletion) by downregulating the expression/function of a synthetic lethal interactor that is required for cancer cell survival. For example, *RNF20*-depleted cancer cells are more sensitive to DNA damage induced by the PARP1 inhibitors olaparib or BMN673 than isogenic control cells [120]. Such synthetic lethal strategies may represent an effective alternative to developing DUB inhibitors, and could be employed to target cancers exhibiting aberrant H2BK120ub1 depletion.

### 3.3. Increased H2BK120ub1 Abundance as an Emerging Cancer Therapeutic Target

Although the immunohistochemical studies described above show that H2BK120ub1 levels are reduced in many cancer types, there are also immunohistochemical studies showing that H2BK120ub1 levels are increased in a subset of cancers [15,88,89]. For example, whereas H2BK120ub1 levels are reduced in triple-negative breast cancers, increased H2BK120ub1 abundance occurs in luminal estrogen-receptor (ER)-positive breast cancers [15]. Perhaps even more important, high H2BK120ub1 levels are associated with worse patient survival in ER-positive breast cancer cases [15]. Furthermore, DNA- and RNA-sequencing data show that multiple cancer types, including colorectal cancer, luminal breast cancer, and ovarian cancer, frequently exhibit gene amplification or overexpression of *RNF20* and/or *RNF40*, and/or deletion (i.e., heterozygous or homozygous deletion) and reduced expression of *USP22* [15,70,91], suggesting that aberrant increases in H2BK120ub1 may also promote oncogenesis. In this regard, high H2BK120ub1 levels and *RNF20/RNF40* expression promote expression of ER target genes [15,88] and proliferation of luminal breast cancer cells [15]. In mixed lineage leukemia (MLL)-rearranged leukemia, *RNF20* expression also promotes cell proliferation, and *RNF20*-mediated H2BK120ub1 is enriched within the body of MLL-fusion target genes and promotes their expression [121]. In addition, H2BK120ub1 plays important roles in DSB repair (see Section 2 and Table 2), and reduced expression and/or function of USP22 prevents timely H2BK120ub1 deubiquitination, which impairs the DSB repair process [75,76]. Thus, reduced *USP22* expression may induce chromosome instability and promote cancer development and progression. Collectively, these recent observations coupled with those detailed above (Section 3.2) suggest that H2BK120ub1 levels must be tightly regulated, as both decreases and increases likely promote oncogenesis. Accordingly, cancer patients with increased H2BK120ub1 abundance may benefit from the development of therapeutic strategies that target overexpression of H2BK120ub1 E3 ubiquitin ligases or exploit reduced DUB activity via synthetic lethality.

## 4. Limitations and Challenges of Therapies Targeting Histone Ubiquitination

Translation of strategies targeting histone ubiquitination into the clinic is currently hampered by four fundamental challenges. Firstly, small-molecule inhibitors against major histone E3 ubiquitin ligases and DUBs are needed, as, to date, clinically relevant inhibitors were only reported for a small subset, including RING1A/RING1B/BMI1. Technical challenges associated with the development of specific E3 ubiquitin ligase or DUB inhibitors (reviewed elsewhere [129,130]) include the lack of suitable assays reproducing physiological conditions [131,132]. In addition, DUB inhibitors directed against the catalytic thiol group are often non-specific as DUB specificity is regulated by factors independent of the catalytic site (i.e., interactions with the ubiquitin and target protein, and allosteric regulation) [133]. However, new in vitro E3 ubiquitin ligase and DUB activity assays that better model the physiological conditions and reduce false-positive rates may expedite the development of histone E3 ubiquitin ligase and DUB inhibitors [131,134,135,136]. In addition, novel cell-based assays enabling high-throughput in vivo drug screening may facilitate the identification of inhibitors with acceptable pharmacological properties [136,137]. Furthermore, better characterizing allosteric regulations of histone E3 ubiquitin ligases and DUBs may highlight new targeting opportunities. For example, the E3 ubiquitin ligase activity of RING1A/RING1B is now targeted with an inhibitor of the allosteric activator BMI1. Similarly, since the deubiquitinating activity of USP22 for H2BK120ub1 functions within the multimeric Spt–Ada–Gcn5 acetyltransferase (SAGA) complex, inhibitors could conceivably be developed that target USP22-interacting partners within the SAGA complex, such as ataxin 7 like 3 (ATXN7L3) [72]. Thus, improved screening technologies coupled with ongoing characterization of enzymes of interest are likely to yield novel inhibitors of histone E3 ubiquitin ligases and DUBs.

Secondly, although targeting histone ubiquitination based on an individual patient’s tumor biology may be considered a form of “targeted therapy”, important concerns remain regarding potential off-target effects. Similar to traditional epigenetics therapies that target DNA methylation or histone acetylation, targeting H2AK119ub1 or H2BK120ub1 will induce genome-wide transcriptional changes affecting hundreds of genes in addition to the loci of interest (Figure 2C) [13,17,61]. The impact of these changes may be largely cell-type-dependent and further modulated by the mutational landscape. This therapeutic concern is supported by a recent report indicating that RNF20 differentially regulates multiple genes in basal-like breast cancer compared to luminal breast cancer. For example, RNF20 represses the oncogene *EZH2* in the basal breast cancer cell line HCC1937, while promoting its expression in the luminal breast cancer cell line MCF7 [15]. Thus, determining whether targeting H2AK119ub1 or H2BK120ub1 in a given context will promote or suppress cancer cell proliferation may be largely unpredictable and require time-consuming, individualized analysis of various cancer subtypes. This may hinder uptake of therapies targeting histone ubiquitination for treatment of additional cancer beyond those for which they are initially approved. In addition, and as with many current treatment approaches, one can expect that, within solid tumors exhibiting high levels of intratumoral genetic heterogeneity, distinct cell populations will respond differently to treatment, and proliferation of certain subpopulations could conceivably be increased by the treatment, rendering it ineffective or promoting resistance. Furthermore, if a treatment is administered systemically, transcriptional reprogramming may also occur within normal cells, and altered expression of oncogenes and tumor suppressors may result in secondary cancers. Therefore, the safety of transcription-focused therapies exploiting histone ubiquitination may be improved by ongoing efforts to design drug-delivery mechanisms that selectively deliver treatments to the targeted cancer cells (e.g., nanoparticle delivery) [138].

Thirdly, most histone ubiquitination sites are regulated by multiple E3 ubiquitin ligases and DUBs (see Table 1 and Table 2), and functional compensation may drive therapeutic resistance. Characterization of each of these enzymes is still ongoing as regards cell-type specificity, temporal dynamic, and impact on cellular processes including transcription and DNA damage repair; thus, the level of functional complementation remains unclear. If certain enzymes are fully functionally redundant, multiple E3 ubiquitin ligases or DUBs would need to be simultaneously inhibited to achieve the required therapeutic benefit. In addition, partially redundant E3 ubiquitin ligases or DUBs, with different substrate affinities or distinct temporal dynamics, may become overexpressed to compensate for target inhibition and underlie treatment resistance.

Finally, as with histone acetyltransferases, it is becoming increasingly evident that histone E3 ubiquitin ligases and DUBs can also target non-histone substrates [69,92,139]. Thus, a therapeutic strategy targeting a specific E3 ubiquitin ligase or DUB may have additional off-target effects, including misregulation of the abundance, cellular localization, and/or function of these non-histone substrates (Figure 2C). These unintended effects may be better understood in the future, as ongoing research efforts seek to identify these non-histone targets.

## 5. Harnessing the Full Potential of Therapies Targeting Histone Ubiquitination to Improve Outcomes of Cancer Patients

The development of therapies targeting histone ubiquitination is at an early stage of development. Broad clinical adoption of these therapies will require substantial research efforts aimed at (1) better characterizing the functions and cell-type specificity of histone E3 ubiquitin ligases and DUBs, (2) understanding the transcriptional impact of manipulating histone ubiquitination, and (3) developing selective inhibitors with appropriate pharmacological properties. In parallel, ongoing efforts to develop more selective epigenome editing tools and synthetic lethal strategies, and to characterize the role of histone ubiquitination in DNA damage repair are likely to accelerate the development of therapies targeting histone ubiquitination with enhanced specificity and efficacy.

Identifying and exploiting synthetic lethal dependencies of cancer cells with aberrant histone ubiquitination will enable the development of therapeutic strategies that bypass the potential pleiotropic off-target effects associated with direct targeting of an aberrantly expressed histone E3 ubiquitin ligase or DUB. Instead, targeting an appropriate synthetic lethal partner with a well-defined impact on a single pathway may be a more predictable and a safer strategy to exploit aberrant regulation of histone ubiquitination in cancers. Moreover, synthetic lethal strategies have the unique benefit of enabling targeting of loss-of-function alterations, which would be relevant for cancer types frequently exhibiting reduced expression of E3 ubiquitin ligases (e.g., *RNF20* or *RNF40*) or DUBs (e.g., *BAP1* or *USP22*).

**Figure 4:**
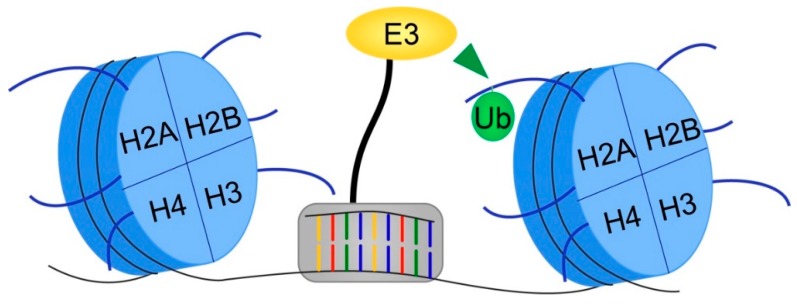
Schematic depicting an epigenome editing tool to target histone ubiquitination. Epigenome editing employs a fusion protein consisting of a DNA sequence-specific recognition domain (gray box) and a functional chromatin-modifying domain, such as a histone E3 ubiquitin ligase, in order to modulate a chromatin post-translational modification at a specific locus.

Beyond synthetic lethal strategies, epigenome editing is an attractive alternative, as it employs fusion proteins combining a sequence-specific DNA recognition domain with the catalytic domain of a chromatin-modifying enzyme to specifically alter chromatin at selected loci (Figure 4) [140]. This technique, currently in its infancy, may enable the development of strategies modulating histone ubiquitination that target a specific locus (or loci) of interest without inducing genome-wide transcriptional changes, or altering ubiquitination levels of E3 ubiquitin ligase and DUB non-histone substrates. Employing this technique will require systematic identification of the critical driver genes aberrantly regulated by histone ubiquitination in a particular cancer type. Nevertheless, developing epigenome-editing effectors to add or remove histone ubiquitination at key loci has strong therapeutic potential that could be applied in many different cancer contexts; however, as with any novel therapeutic strategy, off-target effects would need to be adequately addressed.

Finally, although current histone ubiquitination-based strategies focus predominantly on transcriptional reprogramming of cancer cells, exploiting the role of histone ubiquitination in DNA damage repair may be an effective alternative. Importantly, clinical assessments of epigenetic therapies employing histone deacetylase inhibitors indicate that transcriptional reprogramming may not be the most effective use of these epigenetic therapies, particularly within solid tumors exhibiting genetic and epigenetic heterogeneity [2,141,142]. Importantly, emerging data show that employing histone deacetylase inhibitors to sensitize cancer cells to DNA damage-inducing treatments may be more effective and selective toward cancer cells, and better limit off-target effects and treatment resistance [2,141]. Similarly, exploiting the critical functions of histone ubiquitination in DSB repair may further sensitize cancer cells to existing DNA-damage-inducing therapies and, thus, may be very effective in a combinatorial-based therapeutic strategy. As DSB repair pathways are conserved across cell types, histone ubiquitination-based therapies that target DNA damage repair are predicted to be more broadly applicable than the inherently cell-type-dependent transcription-focused strategies. Furthermore, selective targeting of cancer cells can be achieved by targeting histone ubiquitination marks associated with DSB repair, on which cancer cells are uniquely dependent for survival (i.e., synthetic lethality). For example, upregulation of the H2AK15 E3 ubiquitin ligase RNF168 is critical for the resistance of cancer cells to endogenous or treatment-induced proteotoxic stress [95] and, therefore, RNF168 may be a promising target for a therapeutic strategy exploiting aberrant histone ubiquitination in cancers. Ongoing efforts to characterize both normal and cancer-associated functions of histone ubiquitination within the DNA damage repair pathways may highlight additional targets for the development of DNA damage-centered histone ubiquitination therapies.

## 6. Conclusions

Tremendous progress in our understanding of the function of histone ubiquitination in cancers is supporting the development of therapeutic strategies that aim to reprogram transcription, in order to halt cancer cell proliferation and induce cell death. However, such transcription-based strategies are currently limited by multiple challenges associated with pleiotropic off-target effects that may restrict their use to cancer types with poor prognosis and limited treatment options. These shortcomings are driving the development of new approaches, including epigenome editing tools and synthetic lethal strategies, which aim to increase the specificity, efficacy, and applicability of therapies exploiting histone ubiquitination, to ultimately benefit a broader spectrum and increased number of cancer patients.

## Figures and Tables

**Table 1:** Functions and regulation of histone H2A ubiquitination marks.

Writers ^1^	Erasers	Readers and Function
**H2AK15ub1**		
RNF168 (recruited by RNF8-mediated H1 polyubiquitination) [27,48]	-USP3 [52] -USP51 [53] May also be directly or indirectly regulated by: -USP44 [54]	DNA damage repair: In the vicinity of a DNA double-strand break (DSB), H2AK15ub1 mediates recruitment of 53BP1 (in conjunction with H4K20 di-methylation). 53BP1 acts as a protein scaffold recruiting DNA repair proteins, which modulate DNA end resection and promote error-free repair [47].
**H2AK15 polyubiquitination (K63-linked ubiquitin chains)**		
Initiated by RNF168, elongated by RNF8 [48]	BRCC36 [55]	DNA damage repair: Following DSB, H2AK15 polyubiquitination recruits the BRCA1-A complex (including E3 ubiquitin ligase BRCA1/BARD1), which inhibits DNA end resection [49,50].
**H2AK119ub1**		
-RING1A/RING1B (catalytic subunit of PRC1 complexes) activated by BMI1 [33,34,35] -DZIP3 [56]	-BAP1, activated by ASXL1, ASLX2 or ASLX3 [36] -MYSM1 [57] -USP3 (DNA damage repair) [52] -USP10 (H2A.Z variant) [58] -USP16 (regulation of hematopoiesis) [37] -USP21 [38] -USP22 [59,60]	Transcriptional repression: -Prevents recruitment of the FACT (facilitates chromatin transcription) complex, which blocks transcription elongation [56] -Prevents methylation of gene-activating marks H3K4me2-3 [38] -Recruits chromatin remodeling protein RSF1 [61] -Recruits PRC2 polycomb repressive complexes, which catalyze the repressive mark H3K27me3 [62,63,64] -Deubiquitination by MYSM1 promotes androgen receptor-dependent gene activation [57] DNA damage response: -Represses transcription of DSB-flanking regions [46] -Promotes rapid DSB repair [46]
**H2AK127ub1 and H2AK129ub1**		
BRCA1/BARD1 [29]	USP48 [65]	DNA damage response: Recruits SMARCAD1, which promotes DNA end resection and homologous recombination [51]

^1^ Only E3 ubiquitin ligases, primarily responsible for substrate specificity, are listed.

**Table 2:** Functions and regulation of histone H2B ubiquitination marks.

Writers ^1^	Erasers	Readers and Function
**H2BK34ub1**		
MSL1/MSL2 [66,73]	Unknown	Transcription activation: -Promotes methylation of transcription-activating marks H3K4me3 and H3K79me2 [66] -Promotes conversion of nucleosomes into hexasomes via H2A/H2B dimer eviction [23]
**H2BK120ub1**		
-RNF20/RNF40 [67,68] -MDM2 (H2BK120 activity may be restricted to p53 target genes) [77] -BAF250/Elongin-C/Cullin-2/RBX1 [78]	-USP3 (DNA damage repair) [79] -USP22 (transcription regulation; DSB repair) [60,80] -USP27X (transcription regulation) [72] -USP36 (transcriptional repression of target genes) [81] -USP42 (transcriptional activation of target genes) [82] -USP43 (transcriptional repression of NuRD target genes) [83] -USP44 (transcriptional repression of N-CoR target genes) [84] -USP49 (control of mRNA splicing) [85] -USP51 (transcription regulation) [72]	Transcription activation: -Promotes accessible chromatin conformation [26,86] -Promotes transcription elongation by RNA polymerase II in cooperation with FACT [87] -Promotes methylation of transcription-activating marks H3K4me2-3 (by SET1 complex) [68] and H3K79me1-2 (by DOT1L) [24] Transcriptional repression: -MDM2-mediated H2BK120ub1 may promote transcription repression [77] DNA damage repair: -Recruits effectors of the two major DSB repair pathways, non-homologous end-joining and homologous recombination [25,74] -Removal required for class-switch recombination repair, non-homologous end-joining and homologous recombination [75,76]

^1^ Only E3 ubiquitin ligases, primarily responsible for substrate specificity, are listed.

**Table 3:** Functions of histone H2A and H2B ubiquitination marks in cancer.

Alteration of Writers or Erasers in Cancer	Clinical Development
**H2AK15ub1 and H2AK15 poly-ub**	
Proteotoxic stress (endogenous or treatment-induced) depletes ubiquitin pools available for DNA damage signaling. Overexpression of RNF168 and subsequent alteration of DSB repair processes promotes resistance to proteotoxic stress in cancer cells [95].	Not applicable
**H2AK119ub1**	
BMI1, the activator of the RING1A/RING1B E3 ubiquitin ligase, is overexpressed and promotes cancer cell self-renewal in multiple cancer types, including pancreatic cancer, glioblastoma multiforme, pediatric diffuse intrinsic pontine glioma, colorectal cancer, epithelial ovarian cancer, and acute myeloid leukemia [12,13,14,96,97,98,99,100]. BMI1 promotes self-renewal of leukemic cells in part via H2AK119ub1-mediated repression of the *INK4A/ARF* locus [12]. In glioblastoma and colorectal cancer, the effect of *BMI1* overexpression on cancer cell self-renewal is independent of the *INK4A/ARF* locus and involves repression of distinct genes [13,14].	BMI1 inhibitor PTC-596: -Preclinical studies for acute myeloid leukemia [101] -Phase I for advanced solid tumors (NCT02404480 complete) -Phase Ib in combination with carboplatin/paclitaxel for ovarian cancer patients (NCT03206645) -Phase Ib in combination with radiation for pediatric diffuse intrinsic pontine glioma or high-grade glioma (NCT03605550)
Reduced expression of *BAP1* (DUB) occurs frequently in metastatic uveal melanoma, pleural mesothelioma, and clear-cell renal cell carcinoma [92,102,103,104]. *BAP1* germline mutations are associated with a familial syndrome of predisposition to mesothelioma and uveal and cutaneous melanoma [92,105]. Relevance of aberrant H2AK119ub1 in *BAP1*-deficient cancers is not established.	BAP1-deficient cells may be targeted with an EZH2 inhibitor via a synthetic lethal strategy [106]. EZH2 inhibitor tazemetostat is in phase II clinical trial for patients with BAP1-deficient malignant mesothelioma (NCT02860286).
**H2AK127ub1 and H2AK129ub1**	
BRCA1, which is frequently deficient in breast and ovarian cancer, is a well-established tumor suppressor protein maintaining genome integrity via its multiple roles in DNA damage repair [107]. Recent identification of BRCA1 as the E3 ubiquitin ligase for H2AK127/129 indicates that misregulation of H2AK127/129ub1 may contribute to genome instability in cancer [29].	*BRCA1* deficiency sensitizes cancer cells to synthetic lethal targeting with PARP1 inhibitors [108,109]. Advanced *BRCA1*-deficient ovarian cancers are treated with PARP1 inhibitors such as olaparib [110].
**H2BK120ub1**	
Global loss of H2BK120ub1 is observed in ~70% of breast and colorectal cancers [88,89]. *RNF20* promoter is hypermethylated in breast cancer and *RNF20/RNF40* expression is reduced in seminoma, basal-like breast cancer, and colorectal cancer [15,17,111,112]. *USP22* overexpression is part of the “death from cancer” signature [113] and observed in multiple cancer types, including breast cancer and colorectal cancer [89,114,115,116,117,118,119]	Preclinical study indicates that *RNF20*-depleted cells can be selectively targeted with PARP1 inhibitor via a synthetic lethal strategy [120]
RNF20 promotes breast luminal tumor growth and RNF20-mediated H2BK120ub1 promotes expression of estrogen receptor-α target genes in luminal breast cancer cells [15]. *RNF20* expression is required for proliferation of *MLL* rearrangement-driven leukemia [121]	Not applicable
